# Excessive visit-to-visit glycemic variability independently deteriorates the progression of endothelial and renal dysfunction in patients with type 2 diabetes mellitus

**DOI:** 10.1186/s12882-016-0300-0

**Published:** 2016-07-07

**Authors:** Fang Wei, Xiaolin Sun, Yingxin Zhao, Hua Zhang, Yutao Diao, Zhendong Liu

**Affiliations:** Department of Cardiology, Jinan Central Hospital Affiliated to Shandong University, Jinan, Shandong 250013 China; Cardio-Cerebrovascular Control and Research Center, Institute of Basic Medicine, Shandong Academy of Medical Sciences, NO. 18877, Jingshi Road, Jinan, Shandong 250062 China

**Keywords:** Type 2 diabetes mellitus, Glycemic variability, Endothelial dysfunction, Renal dysfunction

## Abstract

**Background:**

Glycemic variability (GV) creates challenges to glycemic control and may be an independent marker for unfavorable outcome in management of patients with diabetes. This study was designed to investigate the effect of excessive visit-to-visit GV on the progression of endothelial and renal dysfunction in patients with type 2 diabetes mellitus (T2DM).

**Methods:**

Two hundred and thirty nine patients with T2DM, who were recruited from outpatient, completed 48-month follow-up visit. Visit-to-visit GV was calculated by the standard deviation (SD) and coefficient of variation (CV) of serially measured HbA1c and fasting plasma glucose (FPG). Endothelial and renal function was assessed at baseline and end of follow-up.

**Results:**

At end of follow-up, brachial flow-mediated dilation (FMD), nitric oxide (NO), creatinine-based estimated glomeruar filtration rate (eGFR-Cr), and cystatin C-based estimated glomeruar filtration rate (eGFR-Cys C) increased, and endothelin-1 and urine albumin/creatinine ratio (ACR) declined as compared with baseline in overall (*P* < 0.05). The increment of FMD, NO, eGFR-Cr, and eGFR-Cys C and the decrement of endothelin-1 and ACR in first tertile group were significantly greater than those in third tertile group classified by tertile of either SD of HbA1c or SD of FPG. Change percentage of FMD, NO, eGFR-Cr, and eGFR-Cys C were positively, and change percentage of endothelin-1 and ACR were negatively correlated with SDs of HbA1c and FPG, and CVs of HbA1c FPG (*P* < 0.01, respectively). After adjusted for mean HbA1c, mean FPG, baseline demographic, and clinical characteristics, SD of HbA1c and SD of FPG were always statistically correlated with change percentage of FMD, NO, endothelin-1, ACR, eGFR-Cr, and eGFR-Cys C.

**Conclusion:**

Excessive visit-to-visit GV independently deteriorates the progression of endothelial and renal dysfunction in patients with T2DM.

## Background

It is well accepted that type 2 diabetes mellitus (T2DM) is a progressive multisystemic disease accompanied by endothelial [1, 2] and renal dysfunction [3].

Endothelial dysfunction is regarded as a crucial factor in the pathogenesis of vascular disease in diabetes mellitus [4–6]. It is broadly defined as an imbalance between endothelium-dependent vasodilatation and vasoconstriction as well as antithrombotic and prothrombotic factors [7]. Evidences have demonstrated that endothelial dysfunction is closely associated with the development of diabetic microvascular disease including nephropathy and retinopathy in T2DM [8].

Renal dysfunction is one of serious and common clinical microvascular complications in patients with T2DM [3]. It can lead to end-stage renal failure. Glomerular filtration rate (GFR) and urinary albumin excretion (UAE) are both recognized as available indexes of renal function in individuals with or without diabetes [9, 10]. A remarkable meta-analysis, included 45 cohorts and a total of 1,555,332 participants, confirmed that each GFR and UAE is independent predictor of renal outcomes [11].

To reduce the risk of diabetic complications, effective glycemic control is a critical goal of diabetes management [12–15]. But a growing body of evidence reveals that glycemic variability (GV) creates challenges to glycemic control and may be an independent marker for unfavorable outcome in management of patients with diabetes in recent years [12–15]. Moreover, excessive long-term fluctuation, assessed using visit-to-visity GV, in the glycemic control was demonstrated to cause poor outcomes such as macro- and microvascular events and all cause mortality in T2DM patients with the intensive glucose treatment [12]. However, very little information is currently available on correlation between long-term GV, such as visit-to-visit GV, and the progression of endothelial and renal dysfunction in patients with T2DM.

The aim of this study was to investigate the association of excessive visit-to-visit GV with the progression of endothelial and renal dysfunction in patients with T2DM.

## Methods

### Study design and patients

From August 2007, 264 patients with T2DM aged 55 years or older were recruited from outpatient of Cardio-Cerebrovascular Control and Research Center, Institute of Basic Medicine, Shandong Academy of Medical Sciences, China. Patients were ineligible if, in the opinion of the investigator, they met any of the following exclusion criteria: severe hyperglycemia (FPG > 400 mg/dL or 22.2 mmol/L); recent acute serious events such as diabetic ketoacidosis, hyperglycemic hyperosmolar state, severe hypertension (SBP > 170 mmHg and/or DBP > 100 mmHg), secondary hypertension, cerebral stroke, and myocardial infarction in the previous 3 months; heart failure; hemodialysis; abnormal liver enzymes (aspartate aminotransferase and alanine aminotrasferase > 3 times than upper normal range); difficulty with providing informed consent; current participation in another clinical trial. Follow-up visit was conducted every 3 months after baseline visit. All eligible recruited patients were asked to complete 48-month follow-up visit. Guidance of diabetic diet, hypoglycemic therapy, regular exercise, mental adjustment (such as set up confidence to defeat the disease, keep up a positive attitude and optimistic mood.), and self-monitoring were recommended by investigators or specialist doctors in diabetes at each follow-up visit. Eligible patients were asked to strictly execute the guidance. Hypoglycemic agent included metformin, gliclazide, other sulfonylurea, thiazolidinedione, acarbose, glinide, and insulin. HbA1c and fasting plasma glucose (FPG) were monitored at baseline and each follow-up visit in all patients. Hypoglycemia was defined as Zoungas et al. [16] described, namely, a blood glucose level of less than 2.8 mmol/L (50 mg/dL) or the presence of typical symptoms and signs of hypoglycemia without other apparent cause. Severe hypoglycemic episodes were reported with a full description of the event at the time of their occurrence during follow-up visit. Endothelial function [assessed by brachial flow-mediated dilation (FMD), nitric oxide (NO) and endothelin-1 (ET-1)] and renal function [assessed by urinary albumin/creatinine ratio (ACR), estimated GFR based on creatinine (eGFR-Cr) and estimated GFR based on cystatin C (eGFR-Cys C)] were evaluated at baseline and end of follow-up visit.

This study confirmed to good clinical practice guidelines and was conducted in compliance with the “Declaration of Helsinki”. The Research Ethics Committee of Institute of Basic Medicine, Shandong Academy of Medical Sciences approved this study, and written informed consent was obtained from each participant.

### Glycemic parameter measurements and definition of visit-to-visit glycemic variability

HbA1c (%) was detected using the DCA 2000 analyzer (Miles, Diagnostic Division, Elkhart, IN). Finger-sticks from the patients were collected by trained nurses. The DCA 2000 instrument, base on an immunochemical technique, has been proposed for the rapid and simple evaluation of HbA1c. The accuracy and reproducibility of this HbA1c measurement has been certificated and used in clinical practice, as revealed by its good precision and good agreement with the reference system (Diamat™ using high performance liquid chromatography method) [17, 18]. Fasting plasma glucose (FPG) was measured by routine enzymatic laboratory methods using a Hitachi 7600 automated biochemical analyzer (Hitachi, Ltd, Tokyo, Japan). Mean, standard deviation (SD), and coefficient of variation (CV) of each patient’s serial HbA1c or FPG throughout follow-up period were calculated. CV = SD/mean × 100 (%). Visit-to-visit GV was assessed using both SD and CV of HbA1c and FPG.

### Brachial flow-mediated dilation measurement

Brachial flow-mediated dilation (FMD) of reactive hyperemia is known to be endothelium-dependent and a widely accepted noninvasively clinical method for assessing systemic endothelial functions [19, 20]. Brachial FMD has been demonstrated to be markedly abnormal in patients with diabetes and those with diabetic microalbuminuria [21, 22]. In the present study, FMD was evaluated from 08:00 to 09:30 in a quiet and temperature-controlled room (20–25 °C) according to the method described by Thijssen et al. [19]. Participants were demanded to fast for 12 h and discontinue smoking, alcohol, caffeine, tea, anti-histamine, vasoactive medications (including nitrates, angiotensin antagonists, calcium antagonists, and angiotensin-converting enzyme inhibitors), and anti-inflammatory medications for 24 h before measurement performed. After at least 10 min of lying in the supine position, the right brachial artery was scanned over a longitudinal section 3 to 5 cm above the elbow using high-resolution ultrasound (Vivid *i*, GE Medical Systems Ultrasound Israel Ltd.) with a handheld 7.5-MHz transducer (7.5-SPC mechanic sector transducer; GE Medical Systems Ultrasound Israel Ltd.) at rest and in response to increased flow. Increased flow was induced by inflation of a pneumatic tourniquet placed around the forearm to a pressure of 250 mmHg for 5 min, followed by a release. Arterial diameter was measured using M-mode echography during the end-diastolic phase at a fixed distance from an anatomic marker at baseline and 60, 90, and 120 s after cuff deflation. The maximum diameter response from the 3 measurements was used to derive FMD. FMD was calculated with the formula: [(maximum diameter – baseline diameter)/baseline diameter] × 100 %. Measurements were performed by one experienced ultrasonographer, images were recorded on video and later analyzed by the same trained reader who was blinded to angiographic and clinical data. In order to determine the reliability of the measurements, 14 patients were randomly selected for repeated assessment. The intra-observer coefficient of variation for FMD was 3.02 ± 1.64 %.

### Nitric oxide measurement

Nitric oxide (NO) is a crucial endothelium-derived molecule for vascular relaxation. It has been found that disturbances in NO bio-availability can cause endothelial dysfunction, leading to increased susceptibility to hypertension, diabetes mellitus and atherosclerotic lesion progression [23, 24].

Serum concentration of NO was measured indirectly by the quantification of nitrite (NO_2_^−^), a stable metabolite of NO, using Griess assay [25]. Briefly, 100 μl of serum were transferred to a flat-bottom 96-well microtiter plate, then mixed with 50 μl of 2 % sulfanilamide in 5 % HCl solution and 50 μl of 0.1 % N-(1-Naphtyl) ethylendiamine in water sequentially. 100 μl vanadium chloride III 0.8 % was added to each sample and incubate at 37 °C for one hour to reduce nitrate to nitrite. The concentration of nitrite was determined by measuring optical density using an ELISA-reader (Poweam Medical Systems Co., Ltd.) in 540 nm. All reagents were purchased from Sigma (St. Louis, MO, USA). Samples were measured in duplicate and the mean was used for further analyze.

### Endothelin-1 measurement

Plasma concentrations of endothelin-1 (ET-1), an important member of the endothelin family and a marker of endothelial injury, were tested using enzyme-linked immunosorbent assay (ELISA) kits following the manufacturer’s instructions (Bender MedSystems, Vienna, Austria). Minimum detectable concentration was less than 1.0 pg/mL. Intra-assay and inter-assay coefficients of variation were less than 5 %. All samples were measured in duplicate.

### Evaluation of estimated glomerular filtration rate

eGFR-Cr and eGFR-Cys C have the most commonly been used to evaluate renal function. Equations of eGFR combining serum creatinine and cystatin C have been indicated to further improve the precision of GFR estimates [26, 27].

Serum concentration of Cr was determined by Jaffe’s kinetic method using a Hitachi 7600 automated biochemical analyzer. The normal reference range was 50–110 μmol/L. GFR estimation from serum Cr was made using the CKD-EPI equation is: eGFR-Cr = 141 × min (SCr/k, 1)^α^ × max (SCr/k, 1)^-1.209^ × 0.993^Age^ (×1.018, if female), which is considered the best in Chinese population [28]. In the equation, k is 0.7 for females and 0.9 for males, α is −0.329 for females and −0.411 for males, “min” indicates the lesser of SCr/k or 1, and “max” indicates the greater of SCr/k or 1.

Cys C was determined by latex enhanced immunoturbidimetric assay (Mike Biotechnology Co., Ltd., Sichuan, China). Variation was less than 4 % for intra-assay and 6 % for interassay. GFR was carried out using Hoek formula: eGFR-Cys C = −4.32 + 80.35 × 1/CysC in mg/L [29].

### Evaluation of urinary albumin excretion

Albuminuria has been extensively recommended as a major prognostic indicator in individuals with diabetes [30, 31]. UAE was determined on the basis of the urinary ACR. Early morning first void sterile urinary spot samples were collected during the health examination. Urinary albumin and creatinine levels were determined by immunonephelometry and the Jaffe reaction-rate method (Hitachi 7600 automated biochemical analyzer), respectively. And then, ACR was calculated.

### Clinical laboratory measurements

Total cholesterol (TCHO), triglycerides (TG), high-density lipoprotein cholesterol (HDL-c), and low-density lipoprotein cholesterol (LDL-c) were measured by routine enzymatic laboratory methods using a Hitachi 7600 automated biochemical analyzer (Hitachi, Ltd, Tokyo, Japan) at baseline and at annual follow-up visit.

### Statistical methods

Statistical analysis was performed using the SPSS 17.0 statistical software (SPSS 17.0 for Windows, Chicago, IL, USA). Continuous values were expressed as means with SD. Normality of data were evaluated using Kolmogorov-Smirnov test. If not normally distributed, the data were expressed as median with inter-quartile range (IQR, the range between the 25th and 75th percentile). Categorical data were expressed as numbers (percentages). Change percentage was used to represent the changes of FMD, NO, ET-1, ACR, eGFR-Cr, and eGFR-Cys C throughout follow-up period. Change percentage was calculated as follows: [(value at end of follow-up – value at baseline)/value at baseline] × 100 %. Accordance with tertile of mean SD of HbA1c, patients were classified into three groups, namely, first tertile group, second tertile group, and third tertile group. Meanwhile, patients were also divided into first tertile group, second tertile group, and third tertile group by tertile of mean SD of FPG. Comparisons of continuous values among groups were performed using one way analysis of variance (ANOVA) with Bonferroni procedure or Kruskal-Wallis test depending on the normality of data. Categorical data were compared by Chi-square test. According to the normality of data, Student’s paired t-test or Mann–Whitney test was used to detect the differences in FMD, NO, ET-1, ACR, eGFR-Cr, and eGFR-Cys C between baseline and end of follow-up. Pearson or Spearman correlation coefficient was used to measure the strength of association between variables. Backward stepwise multiple linear regression analysis was performed to examine the independently relationships of change percentage of FMD, NO, ET-1, ACR, eGFR-Cr, and eGFR-Cys C with visit-to-visit GV and other variables. In the model, 0.05 was used as cutoff for retention and elimination of variables. Value of two-tailed *P* < 0.05 was considered statistically significant.

## Results

### Baseline demographic and clinical characteristics

**Fig. 1:**
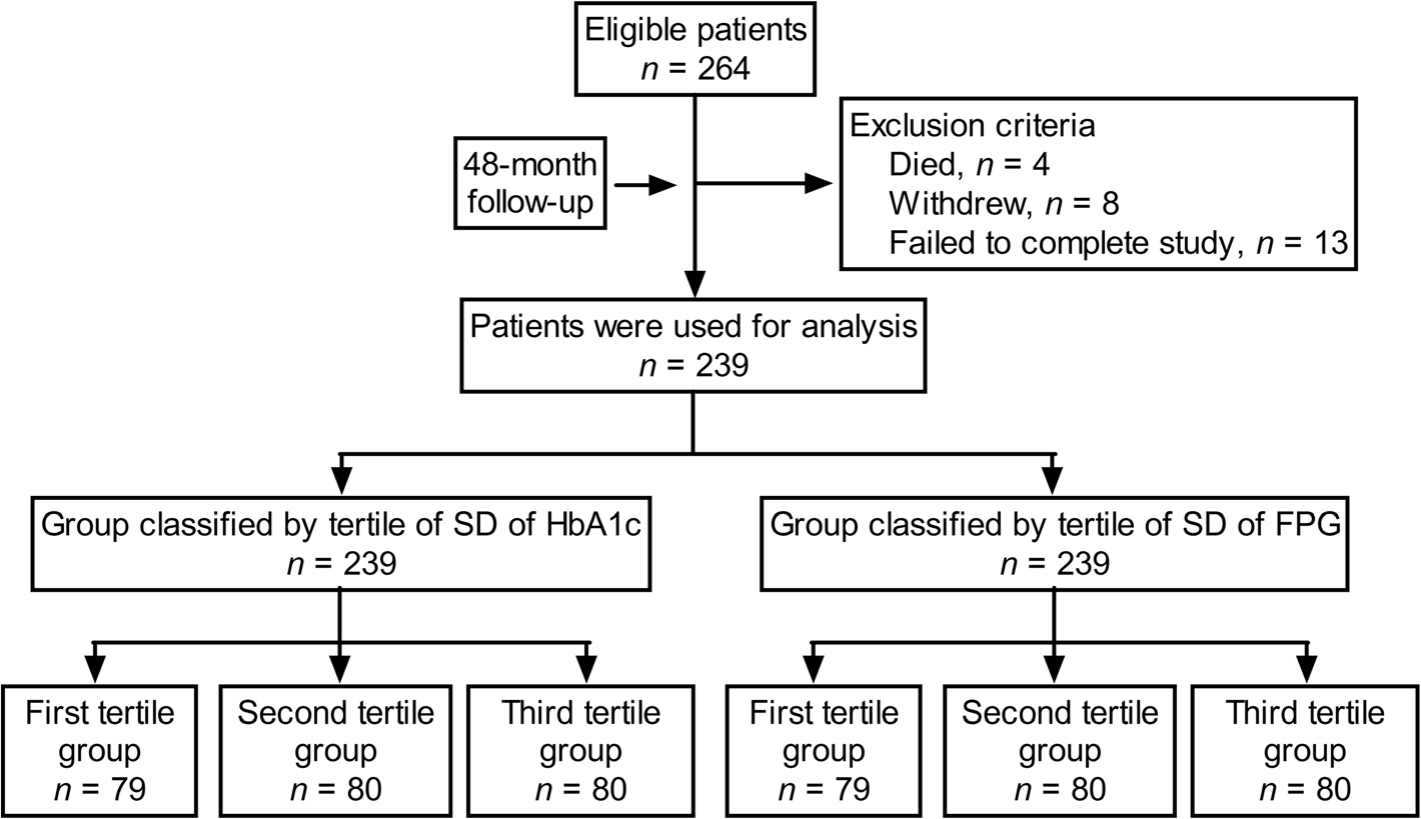
Flow diagram of the study

Figure 1 summarizes the flow diagram of the study. Among 264 patients, 25 patients were excluded for the following reasons: 4 died, 8 withdrew, and 13 failed to complete the study. Finally, 239 participants completed 48-month follow-up visit and were included and used for further analysis.

Baseline demographic and clinical characteristics of participants are summarized in Table 1. There were no statistically significant differences among groups classified by tertile of SD of HbA1c and tertile of SD of FPG with respect to clinical and biochemical variables.

**Table 1:** Baseline demographic and clinical characteristics according to tertiles of SD of HbA1c and SD of FPG

	Classified by tertile of SD of HbA1c				Classified by tertile of SD of FPG			
	First tertile group (*n* = 79)	Second tertile group (*n* = 80)	Third tertile group (*n* = 80)	*P* value	First tertile group (*n* = 79)	Second tertile group (*n* = 80)	Third tertile group (*n* = 80)	*P* value
Age (year)^a^	65.97 ± 5.52	64.36 ± 7.30	64.88 ± 5.74	0.253	65.27 ± 6.09	64.71 ± 6.08	65.23 ± 6.62	0.825
Sex, female (%)^b^	36 (45.57)	42 (52.50)	47 (58.75)	0.252	41 (50.00)	47 (59.49)	37 (47.44)	0.279
BMI (kg/m^2^)^a^	24.43 ± 2.94	23.58 ± 2.71	23.79 ± 2.81	0.147	23.89 ± 2.89	23.44 ± 2.75	24.46 ± 2.79	0.074
SBP (mm Hg)^a^	152.92 ± 11.85	153.01 ± 8.71	156.02 ± 8.99	0.083	152.35 ± 8.16	153.56 ± 9.70	156.03 ± 11.61	0.061
DBP (mm Hg)^a^	83.68 ± 9.54	80.81 ± 7.73	82.92 ± 7.08	0.073	83.06 ± 8.34	82.38 ± 7.35	81.96 ± 8.99	0.701
Heart rate (bpm)^a^	74.82 ± 9.65	76.01 ± 9.09	74.34 ± 8.89	0.498	74.65 ± 10.78	75.30 ± 7.30	75.23 ± 9.31	0.888
Duration of diabetes (years)^c^	3.00 (2.85, 3.25)	3.00 (2.85, 3.27)	3.00 (2.82, 3.33)	0.905	3.00 (2.90, 3.35)	3.00 (2.69, 3.13)	3.00 (2.93, 3.19)	0.342
Current smoking, *n* (%)^b^	8 (10.13)	9 (11.25)	7 (8.75)	0.871	8 (9.76)	8 (10.13)	8 (10.26)	0.994
Current drinking, *n* (%)^b^	5 (6.33)	6 (7.50)	5 (6.25)	0.940	7 (8.54)	3 (3.80)	6 (7.69)	0.442
Hypertension history, *n* (%)^b^	62 (78.48)	60 (75.00)	65 (81.25)	0.631	64 (78.05)	61 (77.22)	62 (79.49)	0.941
FPG (mmol/L)^a^	9.80 ± 1.50	9.80 ± 1.57	10.14 ± 1.54	0.269	9.97 ± 1.43	9.99 ± 1.46	9.78 ± 1.72	0.625
HbA1c (%)^a^	10.01 ± 2.00	10.17 ± 1.53	10.62 ± 1.34	0.061	10.21 ± 1.44	10.19 ± 1.76	10.40 ± 1.77	0.684
TCHO (mmol/L)^a^	5.10 ± 0.99	5.36 ± 0.96	5.39 ± 1.00	0.127	5.41 ± 0.91	5.27 ± 0.96	5.18 ± 1.08	0.332
TG (mmol/L)^a^	1.89 ± 1.11	1.61 ± 0.84	1.73 ± 1.01	0.200	1.65 ± 0.88	1.65 ± 0.88	1.92 ± 1.18	0.139
HDL-c (mmol/L)^a^	1.37 ± 0.45	1.41 ± 0.48	1.38 ± 0.50	0.893	1.38 ± 0.50	1.36 ± 0.41	1.42 ± 0.52	0.768
LDL-c (mmol/L)^a^	3.35 ± 1.15	3.63 ± 1.11	3.67 ± 1.21	0.167	3.70 ± 1.08	3.58 ± 1.03	3.38 ± 1.33	0.215
Hypoglycemic agents, *n* (%)^b^								
Metformin	73 (92.41)	72 (90.00)	75 (93.75)	0.675	74 (90.24)	74 (93.67)	72 (92.31)	0.720
Gliclazide	39 (49.37)	34 (42.50)	35 (43.75)	0.653	37 (45.12)	36 (45.57)	35 (44.87)	0.996
Other sulfonylurea	24 (30.38)	21 (26.25)	22 (27.50)	0.839	22 (26.83)	18 (22.78)	27 (34.62)	0.245
Thiazolidinedione	7 (8.86)	5 (6.25)	8 (10.00)	0.681	11 (13.41)	4 (5.06)	5 (6.41)	0.120
Acarbose	15 (18.99)	15 (18.75)	11 (13.75)	0.613	19 (23.17)	12 (15.19)	10 (12.82)	0.189
Glinide	4 (5.06)	4 (5.00)	3 (3.75)	0.905	5 (6.10)	4 (5.06)	2 (2.56)	0.551
Insulin	9 (11.39)	7 (8.75)	7 (8.75)	0.809	8 (9.76)	8 (10.13)	7 (8.97)	0.969
Other drugs, *n* (%)^b^								
Antihypertension drug	57 (72.15)	56 (70.00)	63 (79.63)	0.425	58 (70.73)	58 (73.42)	60 (76.92)	0.673
Aspirin	56 (70.89)	49 (61.25)	43 (53.75)	0.084	51 (62.20)	47 (59.49)	50 (64.10)	0.836
Statins	11 (13.92)	13 (16.25)	11 (13.75)	0.883	12 (14.63)	12 (15.19)	11 (14.10)	0.982

Results are means ± SDs or medians (25^th^, 75^th^ percentiles) for continuous variables and numbers (percentages) for categorical variables

*SD* standard deviation, *HbA1c* hemoglobin A1c, *BMI* body mass index, *SBP* systolic blood pressure, *DBP* diastolic blood pressure, *FPG* fasting plasma glucose, *TCHO* total cholesterol, *TG* triglycerides, *HDL-c* high-density lipoprotein cholesterol, *LDL-c* low-density lipoprotein cholesterol

^a^compared using ANOVA with Bonferroni procedure

^b^compared using Chi-square test

^c^compared using Kruskal-Wallis test

### Baseline variables of renal and vascular endothelial function

Table 2 shows the baseline variables of renal and vascular endothelial function. ET-1 in third tertile group classified by tertile of SD of HbA1c was significant higher than that in first and second tertile group (*P* < 0.05). Compared with first tertile group classified by tertile of SD of FPG, eGFR-Cys C was lower in third tertile group (*P* < 0.05).

**Table 2:** Baseline variables of renal and vascular endothelial function according to tertiles of SD of HbA1c and SD of FPG

	Classified by tertile of SD of HbA1c				Classified by tertile of SD of FPG			
	First tertile group (*n* = 79)	Second tertile group (*n* = 80)	Third tertile group (*n* = 80)	*P* value	First tertile group (*n* = 79)	Second tertile group (*n* = 80)	Third tertile group (*n* = 80)	*P* value
ACR (mg/mmol)^a^	1.60 (1.40, 2.25)	1.57 (1.10, 2.72)	1.78 (1.20, 2.52)	0.792	1.60 (1.30, 2.14)	1.58 (1.21, 2.50)	1.80 (1.30, 2.80)	0.306
Creatintine (mg/dl)^b^	0.73 ± 0.13	0.74 ± 0.13	0.76 ± 0.13	0.429	0.72 ± 0.12	0.74 ± 0.13	0.77 ± 0.14	0.097
Cystatin C (mg/L)^b^	0.78 ± 0.16	0.83 ± 0.16	0.82 ± 0.16	0.118	0.77 ± 0.16	0.82 ± 0.15	0.83 ± 0.17	0.053
eGFR-Cr (ml · min^−1^ · 1.73 m^−2^)^b^	104.39 ± 26.40	99.89 ± 20.56	96.33 ± 22.21	0.091	102.85 ± 21.48	98.54 ± 22.95	99.19 ± 25.37	0.456
eGFR-Cys C (ml · min^−1^ · 1.73 m^−2^)^b^	102.53 ± 18.30	96.57 ± 19.84	97.28 ± 19.84	0.102	103.26 ± 19.23	97.17 ± 18.16	95.97 ± 19.53^*^	0.036
FMD (%)^b^	8.27 ± 3.77	7.56 ± 3.47	8.08 ± 3.83	0.455	8.07 ± 3.57	8.30 ± 3.87	7.54 ± 3.62	0.413
NO (μmol/L)^b^	61.62 ± 8.90	59.59 ± 9.61	61.47 ± 9.83	0.322	61.09 ± 9.07	62.55 ± 9.60	59.04 ± 9.49	0.061
ET-1 (pg/ml)^b^	40.56 ± 6.63	40.44 ± 7.47	42.84 ± 6.99^*,**^	0.055	40.78 ± 6.77	41.46 ± 6.89	41.60 ± 7.66	0.739

Results are means ± SDs or medians (25^th^, 75^th^ percentiles) for continuous variables and numbers (percentages) for categorical variables

*SD* standard deviation, *HbA1c* hemoglobin A1c, *FPG* fasting plasma glucose, *ACR* albumin/creatinine ratio, *eGFR-Cr* estimated glomerular filtration rate base on creatinine, *eGFR-Cys C* estimated glomerular filtration rate base on cystatin C, *FMD* flow-mediated dilation, *NO* nitric oxide, *ET-1* endothelin-1

^a^compared using ANOVA with Bonferroni procedure

^b^compared using Kruskal-Wallis test

^*^ *P* < 0.05, as compared to first tertile group in the same classified groups

^**^ *P* < 0.05, as compared to second tertile group in the same classified groups

#### HbA1c and FPG profiles and severe hypoglycemic episodes during follow-up period

Table 3 reveals the comparison of HbA1c and FPG profiles and episodes of severe hypoglycemia during follow-up period among three groups classified by the tertile of SD of HbA1c and by the tertile of SD of FPG.

**Table 3:** HbA1c and FPG profiles and severe hypoglycemic episodes during follow-up period

	Classified by tertile of SD of HbA1c				Classified by tertile of SD of FPG			
	First tertile group (*n* = 79)	Second tertile group (*n* = 80)	Third tertile group (*n* = 80)	*P* value	First tertile group (*n* = 79)	Second tertile group (*n* = 80)	Third tertile group (*n* = 80)	*P* value
Mean HbA1c (%)	8.22 ± 1.00	8.33 ± 0.79	8.11 ± 0.56	0.213	8.07 ± 0.65	8.23 ± 0.72	8.35 ± 0.99	0.093
SD of HbA1c (%)	1.41 ± 0.50	2.68 ± 0.36^*^	3.52 ± 0.47^*,**^	<0.001	2.05 ± 0.92	2.64 ± 0.81^*^	2.93 ± 0.99^*^	<0.001
CV of HbA1c (%)	17.66 ± 4.59	32.48 ± 3.68^*^	43.89 ± 4.49^*,**^	<0.001	26.31 ± 9.60	32.21 ± 9.26^*^	32.75 ± 10.63^*^	<0.001
Mean FPG (mmol/L)	6.98 ± 0.97	6.92 ± 0.86	6.70 ± 0.55	0.075	6.76 ± 0.64	6.83 ± 0.74	7.02 ± 0.98	0.106
SD of FPG (mmol/L)	3.49 ± 1.27	3.70 ± 0.91	4.26 ± 1.24^*,**^	<0.001	2.69 ± 0.48	3.68 ± 0.28^*^	5.09 ± 0.95^*, **^	<0.001
CV of FPG (%)	50.10 ± 8.62	53.68 ± 7.80^*^	63.87 ± 8.27^*,**^	<0.001	39.81 ± 5.34	53.87 ± 6.29^*^	72.52 ± 8.56^*, **^	<0.001
Severe hypoglycemic episodes (times)	0.70 ± 0.64	1.09 ± 0.79^*^	1.94 ± 1.63^*,**^	<0.001	1.02 ± 1.08	1.32 ± 1.22	1.40 ± 1.33	0.112

Results are means ± SDs

*SD* standard deviation, *CV* coefficient of variation, *HbA1c* hemoglobin A1c, *FPG* fasting plasma glucose

^*^ *P* < 0.05, as compared to first tertile group in the same classified groups

^**^ *P* < 0.05, as compared to second tertile group in the same classified groups

We compared HbA1c and FPG profiles and episodes of severe hypoglycemia among three groups classified by tertile of SD of HbA1c. SD and CV of FPG and episodes of severe hypoglycemia in third tertile group were higher than those in first and second tertile group (*P* < 0.05). CV of FPG and episodes of severe hypoglycemia in second tertile group were higher than those in first tertile group (*P* < 0.05).

HbA1c and FPG profiles and episodes of severe hypoglycemia were compared among three groups classified by tertile of SD of FPG. SD and CV of HbA1c in second and third tertile groups were higher than those in first tertile group (*P* < 0.05).

#### Vascular parameters and glycemic variability throughout follow-up period

Table 4 demonstrates the change percentage of FMD, NO, and ET-1 from baseline to end of follow-up in overall. In all patients, FMD and NO were significantly increased, and ET-1 was markedly declined at end of follow-up compared to baseline (*P* < 0.05).

**Table 4:** Changes of FMD, NO, ET-1, ACR, eGFR-Cr, and eGFR-Cys C throughout follow-up period

	Total (*n* = 239)	Classified by tertile of SD of HbA1c				Classified by tertile of SD of FPG			
		First tertile group (*n* = 79)	Second tertile group (*n* = 80)	Third tertile group (*n* = 80)	*P* value	First tertile group (*n* = 79)	Second tertile group (*n* = 80)	Third tertile group (*n* = 80)	*P* value
Change percentage of FMD (%)^a^	0.93 (−3.10, 4.92)	4.18 (1.62, 6.14)	−0.20 (−2.94, 4.18)^*^	−2.34 (−4.96, 1.58)^*,**^	<0.001	2.37 (−1.46, 5.69)	0.69 (−3.05, 5.50)	−1.43 (−4.26, 3.27)^*,**^	0.001
Change percentage of NO (%)^a^	1.20 (−6.40, 8.80)	7.61 (2.60, 12.18)	−0.94 (−6.10, 6.08)^*^	−5.45 (−10.00, 2.93)^*,**^	<0.001	4.29 (−5.05, 11.58)	3.00 (−6.85, 8.80)^*^	−2.01 (−7.45, 5.28)^*^	0.002
Change percentage of ET-1 (%)^b^	−0.85 ± 5.16	−3.03 ± 4.61	−0.75 ± 5.17^*^	1.23 ± 4.82^*,**^	<0.001	−2.06 ± 4.65	−0.97 ± 5.41	0.49 ± 5.16^*^	0.006
Change percentage of ACR (%)^a^	−2.09 (−6.11,2.55)	−6.43 (−8.91, −2.53)	−2.09 (−4.78, 0.24)^*^	3.10 (−1.36, 6.28)^*,**^	<0.001	−3.48 (−7.52, −0.50)	−2.09 (−6.06, 2.35)^*^	−0.02 (−3.83, 5.83)^*,**^	<0.001
Change percentage of eGFR-Cr (%)^b^	2.73 ± 6.43	7.49 ± 5.94	1.94 ± 5.35^*^	−1.24 ± 4.65^*,**^	<0.001	3.74 ± 5.90	2.83 ± 6.24	1.61 ± 7.00	0.101
Change percentage of eGFR-Cys C (%)^b^	2.35 ± 5.84	6.64 ± 5.37	1.66 ± 4.89^*^	−1.25 ± 4.24^*,**^	<0.001	3.26 ± 5.34	2.47 ± 5.66	1.32 ± 6.36	0.097

Results are means ± SDs or medians (25^th^, 75^th^ percentiles)

^a^compared using ANOVA with Bonferroni procedure

^b^compared using Kruskal-Wallis test

*P < 0.05, vs. first tertile group

^**^ *P* < 0.05, vs. second tertile group

As with patients classified by tertile of SD of HbA1c, FMD and NO were significant increment in first tertile group (*P* < 0.05), and were significant decrement in third tertile group (*P* < 0.05). ET-1 was significant decrement in first tertile group (*P* < 0.05). Increased percentage of FMD and NO and decreased percentage of ET-1 in first tertile group were obviously greater than those in second and third tertile groups (*P* < 0.05). Increased percentage of FMD and NO and decreased percentage of ET-1 in second tertile group were greater than those in third tertile groups (*P* < 0.05).

As with patients grouped by tertile of SD of HbA1c, increased percentage of FMD in first and second tertile group were higher than that in third tertile group (*P* < 0.05). Increased percentage of NO in first tertile group was higher than that in second and third tertile group (*P* < 0.05). Decreased percentage of ET-1 in first tertile group was greater than that in third tertile groups (*P* < 0.05).

#### Renal function and glycemic variability throughout follow-up period

Changes of renal function and glycemic variability throughout follow-up are shown in Table 4. In all patients, eGFR-Cr and eGFR-Cys C were significantly increased, and ET-1 and ACR were markedly declined at end of follow-up compared to baseline (*P* < 0.05).

As with patients grouped by tertile of SD of HbA1c, eGFR-Cr and eGFR-Cys C were significant increment in first tertile group (*P* < 0.05), and were significant decrement in third tertile group (*P* < 0.05). ACR was significant decrement in first tertile group (*P* < 0.05), and significant increment in third tertile group (*P* < 0.05). Increased percentage of eGFR-Cr and eGFR-Cys C and decreased percentage of ACR in first tertile group were significant greater than those in second and third tertile groups (*P* < 0.05). Increased percentage of eGFR-Cr and eGFR-Cys C and decreased percentage of ACR in second tertile group were greater than those in third tertile groups (*P* < 0.05).

As with patients grouped by tertile of SD of HbA1c, decreased percentage of ACR in first tertile group was significant greater than that in second and third tertile groups (*P* < 0.05). Decreased percentage of ACR in second tertile group was greater than that in third tertile groups (*P* < 0.05).

#### Correlations of change percentage of brachial FMD, NO, ET-1, ACR, eGFR-Cr, and eGFR-Cys C throughout follow-up period with SD of HbA1c, CV of HbA1c, SD of FPG, and CV of FPG in all patients

**Fig. 2:**
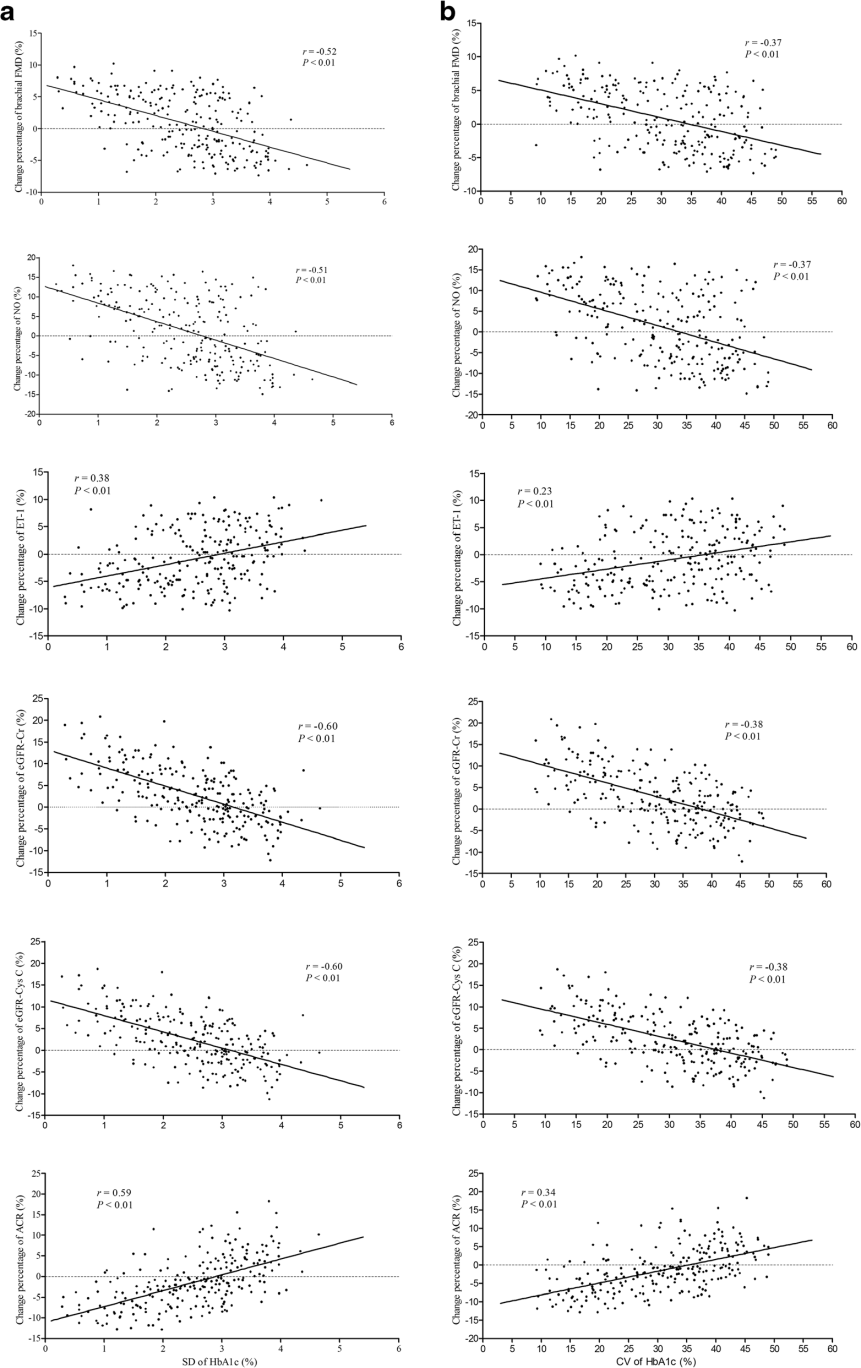
Correlations of SD of HbA1c and CV of HbA1c with change percentage of brachial FMD, NO, ET-1, eGFR-Cr, eGFR-Cys C, and ACR throughout follow-up period. SD of HbA1c was positively correlated with change percentage of ET-1 and ACR, and negatively correlated with change percentage of brachial FMD, NO, eGFR-Cr and eGFR-Cys C (**a**). CV of HbA1c was positively correlated with change percentage of ET-1 and ACR, and negatively correlated with change percentage of brachial FMD, NO, eGFR-Cr and eGFR-Cys C (**b**)

**Fig. 3:**
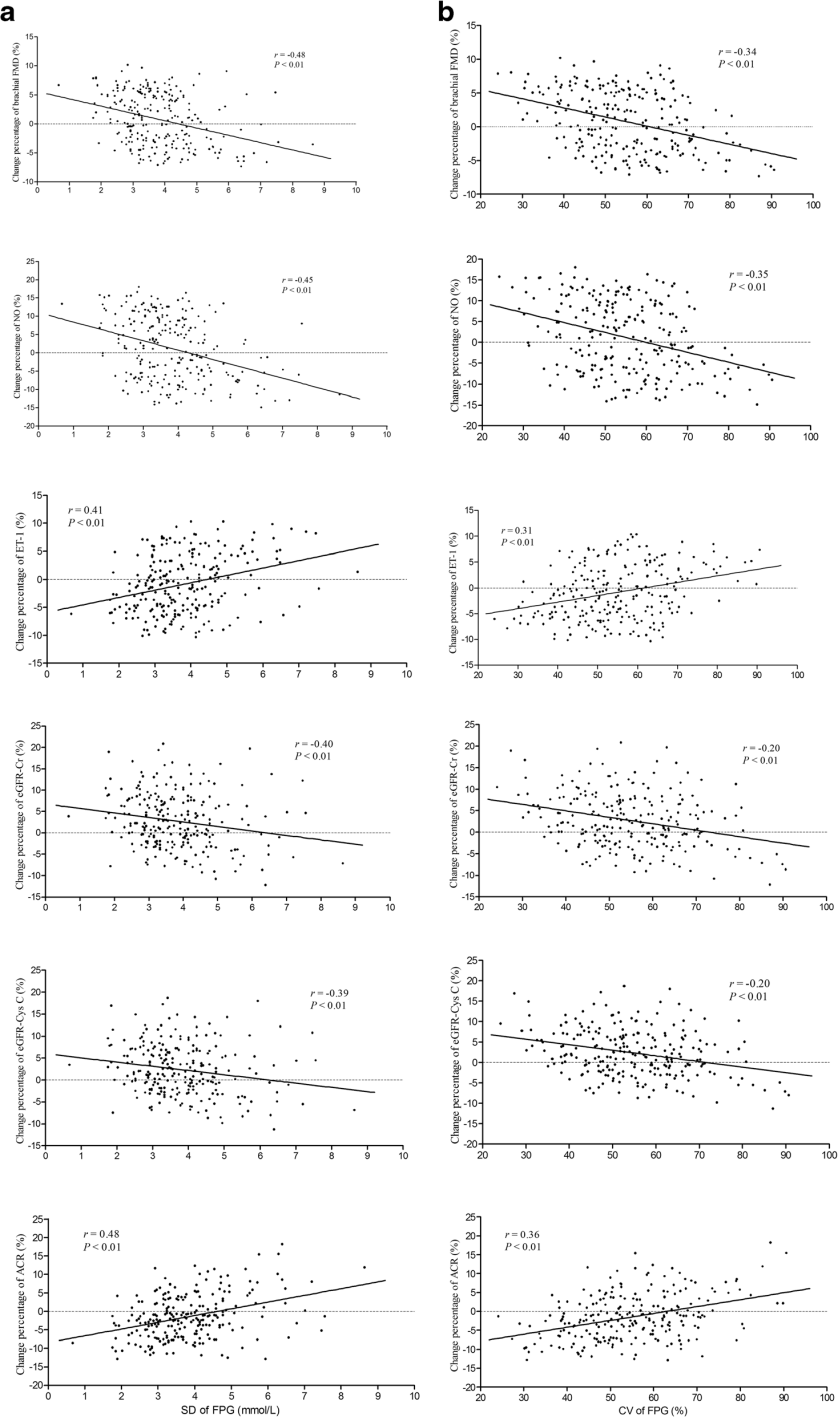
Correlations of SD of FPG and CV of FPG with change percentage of brachial FMD, NO, ET-1, eGFR-Cr, eGFR-Cys C, and ACR throughout follow-up period. SD of FPG was positively correlated with change percentage of ET-1 and ACR, and negatively correlated with change percentage of brachial FMD, NO, eGFR-Cr and eGFR-Cys C (**a**). CV of FPG was positively correlated with change percentage of ET-1 and ACR, and negatively correlated with change percentage of brachial FMD, NO, eGFR-Cr and eGFR-Cys C (**b**)

We assessed the correlations of change percentage of FMD, NO, ET-1, ACR, eGFR-Cr, and eGFR-Cys C with SD of HbA1c (Fig. 2a), CV of HbA1c (Fig. 2b), SD of FPG (Fig. 3a), and CV of FPG (Fig. 3b) throughout follow-up period in total participants.

In all patients, change percentage of FMD, NO, eGFR-Cr, and eGFR-Cys C were remarkable positively, and change percentage of ET-1 and ACR were significant negatively correlated with SD of HbA1c and CV of HbA1c (*P* < 0.01, respectively). Similarly, in all patients, change percentage of FMD, NO, eGFR-Cr, and eGFR-Cys C were remarkable positively, and change percentage of ET-1 and ACR were significant negatively correlated with SD of FPG and CV of FPG (*P* < 0.01, respectively).

#### Change percentage of ACR, eGFR-Cr, and eGFR-Cys C correlate with change percentage of brachial FMD, NO, and ET-1 in total patients

We also evaluated the correlations of change percentage of ACR, eGFR-Cr, and eGFR-Cys C with change percentage of brachial FMD, NO, and ET-1 in total patients.

For change percentage of ACR, there was close negatively correlations with change percentage of FMD and NO, and positively correlation with change percentage of ET-1 (all *P* < 0.001). For change percentage of eGFR-Cr and eGFR-Cys C, there were significant positively correlations with change percentage of FMD and NO, and negatively correlation with change percentage of ET-1 (all *P* < 0.001).

#### Multiple regression analysis

Backward stepwise procedure of multiple linear regression analysis was performed to determine factors independently related to changes of endothelial function and renal function (Table 5). The change percentage of FMD, NO, ET-1, ACR, eGFR-Cr, and eGFR-Cys C was used as dependent variables, respectively.

**Table 5:** Factors possibly related to change percentage of variables of endothelial and renal function throughout follow-up period in total participants

	Change percentage of FMD (%)		Change percentage of NO (%)		Change percentage of ET-1 (%)		Change percentage of ACR (%)		Change percentage of eGFR-Cr (%)		Change percentage of eGFR-Cys C (%)	
	Weight coefficient (95 % CI)	*P* value	Weight coefficient (95 % CI)	*P* value	Weight coefficient (95 % CI)	*P* value	Weight coefficient (95 % CI)	*P* value	Weight coefficient (95 % CI)	*P* value	Weight coefficient (95 % CI)	*P* value
5A. SDs of HbA1c and FPG are included in independent variables in models												
SD of HbA1c	−0.158 (−0.211, −0.105)	<0.001	−0.336 (−0.439, −0.234)	<0.001	0.125 (0.059, 0.190)	<0.001	0.312 (0.242, 0.382)	<0.001	−0.334 (−0.394, −0.274)	<0.001	−0.369 (−0.435, −0.303)	<0.001
SD of FPG	−0.137 (−0.201, −0.072)	<0.001	−0.200 (−0.323, −0.077)	0.002	0.132 (0.053, 0.211)	0.001	0.090 (0.004, 0.176)	0.039	−0.356 (−0.658, −0.054)	0.002	−0.453 (−0.832, −0.074)	<0.001
Mean HbA1c	−0.298 (−0.580, −0.016)	<0.001	--	--	--	--	0.789 (0.006, 1.572)	0.002	--	--	−0.925 (−1.672, −0.178)	0.022
Mean FPG	--	--	−2.306 (−4.294, −0.318)	0.005	--	--	1.524 (0.497, 2.551)	0.009	--	--	−1.188 (−2.369, −0.007)	0.049
Baseline SBP	−0.141 (−0.270, −0.012)	0.006	--	--	--	--	--	--	−0.269 (−0.516, −0.022)	0.013	--	--
Smoking	−0.795 (−1.548, −0.042)	0.025	--	--	0.636 (−0.422, 1.694)	0.047	0.971 (0.009, 1.933)	0.031	−0.466 (−1.755, −0.823)	0.038	−1.335 (−2.248, −0.422)	0.004
Baseline TCHO	--	--	--	--	0.591 (−0.074, 1.256)	0.041	--	--	--	--	−1.925 (−3.572, −0.278)	0.022
Baseline LDL-c	−0.434 (−0.867, −0.001)	0.049	--	--	--	--	--	--	--	--	--	--
5B. CVs of HbA1c and FPG are included in independent variables in models												
CV of HbA1c	−0.165 (−0.219, −0.111)	<0.001	−0.363 (−0.470, −0.256)	<0.001	0.114 (0.048, 0.179)	0.001	0.306 (0.273, 0.376)	<0.001	−0.373 (−0.439, −0.306)	<0.001	−0.339 (−0.400, −0.279)	<0.001
CV of FPG	−0.141 (−0.206, −0.076)	<0.001	−0.162 (−0.293, −0.030)	0.016	0.140 (0.061, 0.219)	0.001	0.092 (0.007, 0.176)	0.034	−0.118 (−0.225, −0.012)	0.029	−0.416 (−0.847, 0.015)	0.041
Mean HbA1c	--	--	--	--	--	--	--	--	--	--	−0.873 (−1.716, −0.030)	0.034
Mean FPG	--	--	−1.045 (−1.902, −0.178)	0.015	--	--	1.530 (0.711, 2.348)	<0.001	−0.530 (−1.012, −0.048)	0.034	−0.966 (−1.257, −0.675)	0.026
Baseline SBP	−0.065 (−0.127, −0.003)	0.040	--	--	--	--	0.121 (0.022, 0.219)	0.017	--	--	--	--
Baseline TCHO	−0.511 (−1.019, −0.002)	0.049	--	--	0.676 (0.045, 1.306)	0.036	--	--	--	--	--	--
Baseline HDL-c	--	--	--	--	−1.139 (−2.229, −0.049)	0.043	--	--	--	--	--	--

Independent variables include age, sex, current smoking, current drinking, duration of diabetes, body mass index, baseline blood press, baseline blood lipids, baseline fasting plasma glucose, baseline HbA1c, mean fasting plasma glucose and HbA1c during follow-up, standard deviation of fasting plasma glucose and HbA1c during follow-up, coefficient of variation of fasting plasma glucose and HbA1c during follow-up, and episodes of severe hypoglycemia over follow-up period

*ACR* albumin/creatinine ratio, *eGFR-Cr* estimated glomerular filtration rate based on creatinine, *eGFR-Cys C* estimated glomerular filtration rate based on cystatin C, *TCHO* total cholesterol, *DBP* diastolic blood pressure, *FPG* fasting plasma glucose, *HbA1c* hemoglobin A1c, *CV* coefficient of variation, *SD* standard deviation

Independent variables included SDs of HbA1c and FPG, age, sex, current smoking, current drinking, duration of diabetes, BMI, baseline blood press, baseline blood lipids, baseline HbA1c, baseline FPG, mean HbA1c and FPG during follow-up, and episodes of severe hypoglycemia over follow-up period. For change percentage of FMD, there were statistically significant results for SD of HbA1c, SD of FPG, mean HbA1c, baseline SBP, smoking, and baseline LDL-c. For change percentage of NO, there were statistically significant results for SD of HbA1c, SD of FPG, and mean FPG. For change percentage of ET-1, there were statistically significant results for SD of HbA1c, SD of FPG, smoking, and baseline TCHO. For change percentage of ACR, there were statistically significant results for SD of HbA1c, SD of FPG, mean HbA1c, mean FPG, and smoking. For change percentage of eGFR-Cr, there were significant results for SD of HbA1c, SD of FPG, mean HbA1c, baseline SBP, and smoking. For change percentage of eGFR-Cys C, there were significant results for SD of HbA1c, SD of FPG, mean HbA1c, mean FPG, smoking, and baseline TCHO.

Independent variables included CVs of HbA1c and FPG, age, sex, current smoking, current drinking, duration of diabetes, BMI, baseline blood press, baseline blood lipids, baseline HbA1c, baseline FPG, mean HbA1c and FPG during follow-up, and episodes of severe hypoglycemia over follow-up period. For change percentage of FMD, there were statistically significant results for CV of HbA1c, CV of FPG, baseline SBP, and baseline TCHO. For change percentage of NO, there were statistically significant results for CV of HbA1c, CV of FPG, and mean FPG. For change percentage of ET-1, there were statistically significant results for CV of HbA1c, CV of FPG, baseline TCHO, and baseline HDL-c. For change percentage of ACR, there were statistically significant results for CV of HbA1c, CV of FPG, mean FPG, and baseline SBP. For change percentage of eGFR-Cr, there were significant results for CV of HbA1c, CV of FPG, and mean FPG. For change percentage of eGFR-Cys C, there were significant results for CV of HbA1c, CV of FPG, mean HbA1c, and mean FPG.

Importantly, from the results of the backward stepwise regression analysis, only SDs of HbA1c and FPG and CVs of HbA1c and FPG were always statistically and independently correlated with change percentage of FMD, NO, ET-1, ACR, eGFR-Cr, and eGFR-Cys C.

## Discussion

This is the first study to explore the association of long-term GV, using both visit-to-visit variabilities of HbA1c and FPG, with the progression of endothelial and renal dysfunction in patients with T2DM. Results of our study demonstrated those (1) excessive visit-to-visit GV associated endothelial dysfunction and renal dysfunction; (2) visit-to-visit GV was statistically correlated with the progression of endothelial dysfunction and renal dysfunction independent of mean HbA1c and FPG, (3) progression of renal dysfunction significant correlated with progression of endothelial dysfunction in T2DM patients.

Effective glycemic control is a critical goal of diabetes management to reduce the risk of diabetic complications [13, 14]. In our study, both endothelial function and renal function were improved along with the decline in HbA1c and FPG during follow-up in patients with T2DM.

However, accumulating evidences demonstrated that there was an independent association between higher variability in glycemic control and worsened outcomes in patients with diabetes [12, 32, 33]. Long-term fluctuations in glycemia, in contrast to short-term glucose instability, contribute to the development of microvascular complications in type 1 diabetes [33]. Kilpatrick and colleagues [33] reported that excessive long-term variability in HbA1c adds to the mean value in predicting diabetic nephropathy and retinopathy. In the observational Finnish Diabetic Nephropathy (FinnDiane) Study [34], GV (assessed by SD of serial measurements of A1c from baseline to follow-up) predicts the development and progression of incipient and overt renal disease in T1DM patients. In T2DM patients, long-term variability in HbA1c is independently associated with the development of microalbuminuria [35].

In consistent with previous studies, at least to some extent, our results revealed that excessive visit-to-visit GV was not only closely associated with the progression of renal dysfunction, but also associated with the progression of endothelial dysfunction independent of mean HbA1c and FPG. Brachial FMD, NO, eGFR-Cr and eGFR-Cys C increased, and ET-1 and ACR declined in first tertile GV group, which classified by tertile of SD of either HbA1c or FPG. On the contrary, brachial FMD, NO, eGFR-Cr and eGFR-Cys C declined, and ET-1 and ACR increased in third tertile GV group. Excessive visit-to-visit GV may be an important risk factor of progression of endothelial dysfunction and renal dysfunction in T2DM patients.

HbA1c, which reflects the 2-to-3 month average endogenous exposure to glucose including postprandial spikes in the blood glucose level and has low intraindividual variability, have become established parameter of long-term glycemic control in diabetics [36]. For this purpose, several analytical methods have been developed. In the present study, HbA1c was evaluated using the DCA 2000 instrument. The DCA 2000 instrument, base on an immunochemical technique, has been proposed for the rapid and simple evaluation of HbA1c. The accuracy and reproducibility of this HbA1c measurement has been certificated and used in clinical practice, as revealed by its good precision and good agreement with the reference system (Diamat™ using high performance liquid chromatography method) [17, 18].

SD is a widely used method to assess variability, although it is a measure of dispersion rather than a measure of GV [37]. SD has been shown a linear relationship with mean glucose. When SD is corrected for the mean and CV is obtained, the linear relationship is largely disappeared [37]. However, Buscemi and colleagues [38, 39] evaluated GV using CV measured by continuous glucose monitoring. They found that CV of glucose was closely related to endothelial dysfunction and atherosclerosis even in non-diabetic subjects. Variability was evaluated by both SD and CV in the present study. Our results agreed with DeVries [37] and there were some differences with Buscemi [38, 39]^.^ Reason of the differences between our study and Buscemi’s may be as follows: Buscemi’s assessed short-time GV using continuous glucose monitoring, yet we evaluated visit-to-visit GV.

As endothelial dysfunction is a systemic disorder [40], it is deemed that endothelial dysfunction plays a crucial role in the pathogenesis of diabetic nephropathy [41]. On the other hand, renal dysfunction is regarded to be an important factor in causing endothelial dysfunction in the systemic vasculature [42, 43]. In the present study, changes of endothelial function strongly related to changes of renal function. However, it was unclear whether change of renal function was a cause or a consequence of change of endothelial function.

Episode of severe hypoglycaemia is a common side effect of hypoglycemic therapy in patients with T2DM and presents a barrier to achieving optimal diabetes management [44]. Hypoglycemia not only can impact patient quality of life, but also is regarded as a contributor to renal dysfunction, cardiovascular events and mortality [44, 45]. Evidences had shown that excessive GV in on-treated patients with diabetes implies that there were more hypoglycemic and/or hyperglycemic episodes, although the average HbA1c levels are desirable [35]. In the present study, we observed that episodes of severe hypoglycemia in higher SD of HbA1c groups were more than those in lower SD of HbA1c groups. With regard to the differences in episodes of severe hypoglycemia among three groups, it is possible affecting the relationship between GV and the progression of endothelial and renal dysfunction in patients with T2DM. However, GV was still significantly associated with the progression of endothelial and renal dysfunction in patients after adjusted episodes of severe hypoglycemia.

Yet, there were several limitations that need to be addressed to our study. First, only a small number of subjects were enrolled in our study. Large-scale and multicenter prospective investigations are needed in the future. Second, as above described, it was unclear of association between change of renal function and change of endothelial function. It needs further study, even in vitro, in the future to clarify the association. Third, 25 patients exclusion from the analyses might have introduced a bias in this study.

## Conclusions

In conclusion, our study documents that excessive visit-to-visit GV is associated with the progression of endothelial and renal dysfunction in patients with T2DM. Controlling excessive visit-to-visit GV may be an important factor to improve endothelial and renal function in management and treatment of patients with T2DM.

## Abbreviations

T2DM: Type 2 diabetes mellitus
GV: Glycemic variability
T1DM: Type 1 diabetes mellitus
HbA1c: hemoglobin A1c
FPG: Fasting plasma glucose
SD: Standard deviation
CV: Coefficient of variation
FMD: Flow-mediated dilation
NO: Nitric oxide
ET-1: Endothelin-1
GFR: Glomerular filtration rate
UAE: Urinary albumin excretion
eGFR: Estimated glomerular filtration rate
Cr: Creatinine
Cys C: Cystatin C
eGFR-Cr: Estimated glomerular filtration rate based on creatinine
eGFR-Cys C: Estimated glomerular filtration rate based on cystatin C
ACR: Albumin/creatinine ratio
TCHO: Total cholesterol
TG: Triglycerides
HDL-c: High-density lipoprotein cholesterol
LDL-c: Low-density lipoprotein cholesterol
